# Exploring Predictors of Type 2 Diabetes Within Animal-Sourced and Plant-Based Dietary Patterns with the XGBoost Machine Learning Classifier: NHANES 2013–2016

**DOI:** 10.3390/jcm14020458

**Published:** 2025-01-13

**Authors:** Adam C. Eckart, Pragya Sharma Ghimire

**Affiliations:** Department of Health and Human Performance, Kean University, Union, NJ 07083, USA; pghimire@kean.edu

**Keywords:** animal-sourced foods, plant-based diets, chronic disease, animal protein, animal fat, plant protein, plant fat, machine-learning, confounding, lifestyle

## Abstract

**Background/Objectives**: Understanding the relationship between dietary patterns, nutrient intake, and chronic disease risk is critical for public health strategies. However, confounding from lifestyle and individual factors complicates the assessment of diet–disease associations. Emerging machine learning (ML) techniques offer novel approaches to clarifying the importance of multifactorial predictors. This study investigated the associations between animal-sourced and plant-based dietary patterns and Type 2 diabetes (T2D) history, accounting for diet–lifestyle patterns employing the XGBoost algorithm. **Methods**: Using data from the National Health and Nutrition Examination Survey (NHANES) from 2013 to 2016, individuals consuming animal-sourced foods (ASF) and plant-based foods (PBF) were propensity score-matched on key confounders, including age, gender, body mass index, energy intake, and physical activity levels. Predictors of T2D history were analyzed using the XGBoost classifier, with feature importance derived from Shapley plots. Lifestyle and dietary patterns derived from principal component analysis (PCA) were incorporated as predictors, and high multicollinearity among predictors was examined. **Results**: A total of 2746 respondents were included in the analysis. Among the top predictors of T2D were age, BMI, unhealthy lifestyle, and the ω6: ω3 fatty acid ratio. Higher intakes of protein from ASFs and fats from PBFs were associated with lower T2D risk. The XGBoost model achieved an accuracy of 83.4% and an AUROC of 68%. **Conclusions**: This study underscores the complex interactions between diet, lifestyle, and body composition in T2D risk. Machine learning techniques like XGBoost provide valuable insights into these multifactorial relationships by mitigating confounding and identifying key predictors. Future research should focus on prospective studies incorporating detailed nutrient analyses and ML approaches to refine prevention strategies and dietary recommendations for T2D.

## 1. Introduction

The relationship between diet and disease has long been a significant interest in public health and nutrition. Dietary patterns and nutrient intake have wide-ranging implications for general health, and understanding the influence of specific nutrients on disease risk is vital for effectively formulating dietary guidelines. The existing literature that compares dietary patterns emphasizes the differences between those focused on plant-based foods (PBFs) and animal-sourced foods (ASFs) in relation to the prevention and management of cardiometabolic diseases, including cardiovascular disease (CVD) and type 2 diabetes (T2D). Many cohort studies show different cardiometabolic diseases and mortality outcomes in association with the intake of plant-based or animal-sourced fat and protein [1,2,3,4,5]. One popular method used in observational studies to demonstrate these effects involves a statistical procedure in which risk ratios are calculated for isocaloric substitutions of one nutrient type for the other [2,3,6]. For example, in one study, replacing 5% of energy from animal fat with 5% from plant fat was associated with reduced overall mortality and CVD mortality [2]. However, this procedure can introduce bias by decoupling subject characteristics from nutrient intakes [7]. In other words, statistical methods that adjust risk ratios based on increases in specific nutrients while holding the total calorie intake constant do not isolate the effect of nutrient proportions alone. They also alter the composition of the sample used in each analysis. Thus, the new risk ratios reflect a different sample of individuals whose disease risk is affected by many other factors besides dietary composition, such as age, obesity status, socioeconomic status (SES), race/ethnicity, or access to healthcare, to name a few. Since dietary patterns are often linked to many health factors, this risk analysis method is problematic, as it may not fully account for healthy user bias, even after adjustments for common confounders [7]. Likewise, individuals with unhealthy lifestyles typically engage in other risk-increasing behaviors or present with multiple comorbidities that may not be accounted for or adjusted for during disease risk analysis, leading to residual confounding.

Lifestyle behaviors and individual characteristics, including physical activity, total energy intake, BMI, sedentary behavior, and age, have an outsized effect on cardiometabolic disease outcomes and are highly interrelated [8]. In many studies, the association between increased risk for cardiometabolic diseases and higher total and animal protein intake compared to plant protein intake applies only to those with unhealthy lifestyle factors [3,6,9,10,11,12]. Adjustments for variables such as BMI, alcohol consumption, physical activity, and smoking often result in attenuation of associations between animal-sourced food intake and disease or mortality outcomes. For example, animal protein was associated with higher incident T2D in the Melbourne Collaborative Cohort Study, while plant protein was inversely associated [6]. However, higher animal protein intake was also positively associated with BMI and fat intake and inversely associated with SES, physical activity, fiber, and vitamin intake. In contrast, plant protein intake was linked to higher physical activity, fiber, and vitamin intake and was inversely associated with BMI and smoking. The inverse association between plant protein intake and T2D attenuated after adjustment for lifestyle factors, fiber, magnesium, vitamin intakes, sodium, and saturated fatty acids [6].

Similarly, in a recent study using data from the National Health and Nutrition Examination Survey (NHANES), the effect of an unhealthy lifestyle characterized by high BMI, higher alcohol consumption, higher total energy, less overall protein intake, higher sugar intake, low physical activity, higher likelihood of smoking, and low participation in other health behaviors (e.g., dental visits and fiber intake) on CVD history was over 2.5 times higher than the effect of red meat intake alone [11]. Moreover, red meat eaters had unfavorable lipid profiles and less physical activity and were more likely to smoke compared to white meat eaters. Evidence from these studies suggests an inexorable link between lifestyle, dietary patterns, and disease outcomes. Consequently, high intake of specific nutrients within hypercaloric diets could be linked to cardiometabolic disease status, irrespective of purported nutrient-disease interactions [13].

Due to the difficulty in isolating the effects of nutrient intakes, links between dietary patterns and chronic diseases would seem paradoxical if other lifestyle factors were not emphasized. For example, in the Shanghai Women’s Health Study, diets higher in dairy were associated with lower T2D risk and a lower likelihood of smoking and alcohol consumption. In contrast, diets higher in plant-based foods were associated with lower SES and higher T2D risk [12]. A prospective study of over 43,000 people found that diets higher in meat consumption compared to those higher in fruits and vegetables were associated with a higher risk of T2D in non-smokers but not in those with a history of smoking [14]. Another prospective study of over half a million people reported that white meat intake was associated with higher CVD risk in former smokers. In contrast, processed meat intake was linked to lower risk among those who never smoked [15].

Confounding issues in nutrient-disease research warrant changes in the methodological approach to account for bias and to identify relevant modifiable risk factors. Traditional statistical approaches cannot effectively handle the challenges posed by high-dimensional data, non-linear relationships, and interactions prevalent in nutritional epidemiology. Developments in machine learning (ML) techniques offer new approaches to reducing model overfit and improving predictive power. They also provide greater flexibility in dealing with high-dimensional and deeply interrelated predictors, which are very common in nutritional epidemiology. These methods enable the examination of large and complex datasets, managing multicollinearity, providing robust estimates, or selecting the most relevant predictors. Utilizing these advanced methods can significantly improve the identification of risk factors and enhance dietary recommendations through a better understanding of diet–disease relationships.

T2D is a global public health issue directly linked to dietary and lifestyle factors, making it an ideal health outcome for analyzing multifactorial predictors using ML techniques. Many studies have compared the performances of various ML models in predicting T2D, with gradient-boosted decision trees among the most used and top-performing, as determined by accuracy and area under the receiver operating characteristic curve (AUROC) metrics [16]. Gradient-boosted decision trees are innovative machine learning algorithms that address issues related to predictive modeling, such as overfit or bias, by combining weaker models, correcting errors, and updating predictions. Gradient boosting minimizes the log loss (for classification models) and the size of the tree, improving interpretability [17]. XGBoost is an extreme gradient-boosting decision tree algorithm that utilizes lasso (L1) and ridge (L2) regularization to prevent overfitting and provides feature importance outputs. Regularization adds a penalty (λ) term to the loss function, reducing coefficients for less relevant predictors [17]. This is especially useful when high multicollinearity exists, which may obscure the true relationships between predictors and the outcome. While L2 reduces feature weights near zero, L1 reduces insignificant features to zero, retaining only the most relevant predictors. The degree of L1 and L2 regularization can be optimized during the ML process to improve accuracy.

ML predictive modeling for T2D is still nascent. The feature sets used in these models are heterogeneous, making comparing feature selection across studies challenging. However, in a meta-analysis of 90 studies on the performance of 18 different models, models that included lifestyle, socioeconomic, and diagnostic data were more accurate overall [16]. In another study, lasso regression was used to select relevant predictors of T2D from a population-based cohort that included 2012 adult men and women. Smoking and waist circumference were among the most important lifestyle factors, increasing the odds of T2D incidence by over 65% and 5% per cm, respectively [18]. In a different study, predictors of fasting blood glucose in 650 participants, 270 of whom had T2D, were compared using basic linear, ridge, and lasso regression [19]. There were high variance inflation factors (VIFs), a measure of multicollinearity for total cholesterol and LDL cholesterol, and a moderate VIF for triglycerides. However, there was an agreement among ML models for age, BMI, and gender as significant predictors of T2D. Another study evaluated the predictive performance of five different ML models in predicting T2D history employing data from NHANES. The top three predictors in order of importance were sleep, energy intake, and age, with an AUROC of 83% and an 82% sensitivity [20]. Similarly, a study compared the importance of T2D predictors across logistic regression, an artificial neural network, and decision tree predictive models [21]. All three models achieved moderately high sensitivity, ranking lifestyle factors, SES, and health-related behaviors among the most important predictors.

Given the limited research using gradient-boosted decision tree models to investigate the relationships between animal-sourced and plant-based diets, lifestyle factors, and T2D, we aim to address this gap. Our analysis will focus on common predictors of T2D history typically found in nutrient-disease studies. To add another layer of control, we will propensity score-match ASF and PBF dietary patterns on key confounders. Additionally, we will assess the predictive power of the XGBoost algorithm and investigate how effectively this method can minimize noise from complicated, interrelated data.

## 2. Materials and Methods

### 2.1. Data and Sample Extraction

NHANES is a nationally representative survey of US civilians. Estimates were calculated using combined-cycle case weights following NHANES guidance to address variations in selection probabilities, non-response, missing data, and sub-sample datasets [22]. Data were collected and combined from 2013 to 2016, including age and gender, body composition, physical activity, smoking status, individual food intake, T2D history, T2D medication use, macro-nutrient intake, serum lipids, and metabolic markers (Table S1).

#### Inclusion and Exclusion Criteria

**Figure 1:**
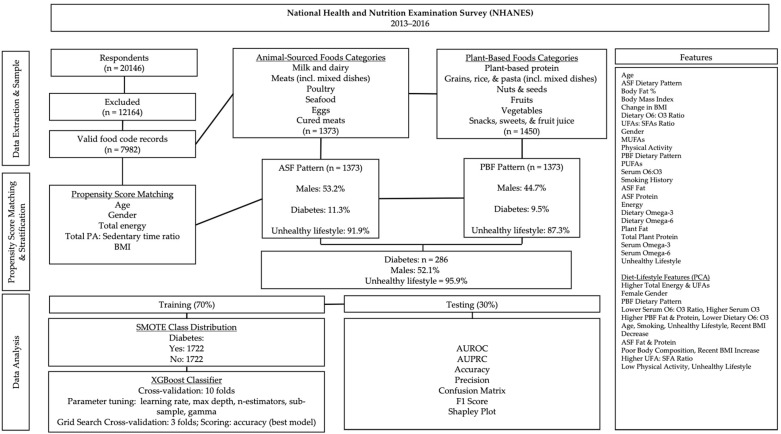
Data processing and analysis overview. Subpopulation percentages are weighted.

Respondents with missing case weights, those following a special diet, individuals with serious disabilities, those taking medication for type I diabetes, those on glucagon-like peptide-1 receptor agonists (GLP-1RAs), participants with a history of bariatric surgery, or those currently pregnant or lactating were excluded from the sample (Figure 1). To enhance generalizability, address the heterogeneity in the relationship between dietary variables and T2D, improve diet–lifestyle pattern analysis, and strengthen propensity score-matching between diet groups, the sample included children and adults aged 16 to 80 years.

### 2.2. Dietary Groups

The NHANES dietary survey records food intakes using USDA food codes. USDA food codes recorded during the dietary interview were classified into broader food categories using the What We Eat in America (WWEIA) food groups (Figure 1). Respondents were selected for the ASF dietary pattern if the percentage of total calories from ASFs was greater than zero. Respondents recording food intake only from plant-based food groups were selected for the PBF dietary pattern.

#### Propensity Score-Matching

After dietary pattern stratification, respondents were propensity score-matched on age, gender, BMI, total caloric intake, and the ratio of physical activity to sedentary time (PAX) without replacement and a 0.5 match tolerance. All variables included in the analyses were examined for normality via the Kolmogorov–Smirnov test (*p* ≤ 0.05).

### 2.3. T2D Cases

Respondents were categorized as having T2D if they answered “Yes” to the question, “Doctor told you have diabetes” and met at least one of the following criteria: glycohemoglobin of 6.5% or higher, current use of T2D medication (insulin or pills) at the time of the survey, or a plasma fasting glucose of ≥126 mg/dL. Gestational or borderline T2D cases were not included in the analysis.

### 2.4. Serum Metabolic Markers and Derived Variables

To address diet quality related to dietary fat, we derived variables, including the ratio of dietary omega-6 (ω6) fatty acid (FA) to omega-3 (ω3) FAs, the serum ω6FAs: ω3FAs ratio, and the ratio of total unsaturated fatty acids (UFAs) to saturated fatty acids (SFAs) [PUFAs + MUFAs/SFAs]. The UFAs: SFAs ratio variable is based on the National Cancer Institute Healthy Eating Index (HEI) scoring standard of ≥2.5 [23].

Evidence suggests that the ratios of dietary and serum ω6FAs: ω3FAs are significant markers of dietary fat quality, which impacts overall inflammation and metabolic health [24,25]. ω3FAs have been shown to exert anti-inflammatory effects, whereas a disproportionately high intake of ω6FAs relative to ω3FAs may exacerbate inflammation. An imbalance in this ratio has been associated with an increased risk of T2D [24,25].

High-sensitive c-reactive protein (hs-CRP) is a biomarker of systemic inflammation, which plays an important role in the pathogenesis of T2D and CVD. Elevated hs-CRP levels have been associated with insulin resistance and an increased risk of developing T2D [26]. By including hs-CRP in the analysis, this study can evaluate the inflammatory responses associated with different dietary patterns and those characterized by varying ω6FA: ω3FA ratios.

Insulin levels directly reflect insulin resistance and pancreatic function. High insulin levels indicate reduced insulin sensitivity, which can lead to T2D. Evaluating insulin levels alongside serum fatty acids, dietary patterns, and hs-CRP provides comprehensive insights into the metabolic associations of T2D.

To address the associations of recent lifestyle changes, we calculated the change in BMI from one year before the survey. To analyze the effect of an unhealthy lifestyle, we categorized respondents based on the presence of at least one unhealthy lifestyle factor, including obesity (≥30 kg/m^2^), smoking history, 1-SD increase in BMI within the past year, or less than 30 min of daily recreational physical activity at any intensity.

### 2.5. Statistical Analyses

To examine multicollinearity, linear regression was used to derive each predictor’s variance inflation factor (VIF). We used principal component analysis (PCA) to analyze diet–lifestyle patterns for those with confirmed T2D. Principal components were rotated via direct oblimin (∆=0) to maximize interpretability. Regression factor scores from each component were added as new features. Variable patterns loading on each component were characterized according to the strength of partial correlations for each predictor in the pattern matrix.

The XGBoost classifier was configured for optimization using a parameter grid for learning rate, n-estimators (the number of trees in the ensemble), max depth (the number of allowable branches), subsample (the percentage of the sample used for training ensemble trees), and gamma value (the regularization parameter that controls the number of tree splits). Training was performed on 70% of the data and tested on 30%.

The classes in the training dataset were balanced using the Synthetic Minority Oversampling Technique (SMOTE). We used a random state of 42 to generate synthetic samples of the T2D-positive class by interpolating between observed cases. In this way, there is equal representation for the classes in the resampled training set, which overcomes the problem of class imbalance. The resampled data were then used in model training and optimization to improve predictive performance and equity across both classes. Sensitivity and accuracy analyses were performed via AUROC and precision-recall curves (AUPRC). Shapley Additive Explanation (SHAP) plots were generated to explain the contribution of each predictor to T2D status in order of importance. Data aggregation, transformation, cleaning, and propensity score matching were performed in IBM SPSS (version 29.0); PCA and XGBoost analyses were performed in Python (version 3.9.6).

## 3. Results

The diet-matched sample included 1373 respondents in each dietary group. The proportion of males in the ASF and PBF groups was 53.2% and 44.7%, respectively. The median age of those in the PBF pattern was 48 years compared to 47 years in the ASF pattern (Table 1). The percentage of respondents with T2D was 11.3% and 9.5% in the ASF and PBF groups, respectively. The median total energy intake in the ASF pattern was 7% higher than in the PBF pattern. Those in the ASF pattern consumed a median intake of 14.71% of their total calories from ASFs. In contrast, those in the PBF pattern consumed 9% of their total calories from PBFs.

Median dietary intakes of cholesterol, total ω3FAs, total ω6FAs, MUFAs from PBFs, PUFAs from PBFs, total plant protein, total plant fat, overall protein, overall fat, total MUFAs, total PUFAs, and saturated fat were higher in the ASF pattern. Conversely, carbohydrates, the dietary ω6FAs: ω3FAs ratio, and fiber were higher in the PBF pattern. The ASF group had a higher intake of MUFAs and PUFAs from animal sources than from plant sources. The UFA: SFA ratio was similar between groups.

Serum values of HDL-C, LDL-C, glycohemoglobin, total cholesterol, triglycerides, total serum ω3FAs, total serum ω6FAs, and plasma fasting glucose were similar. However, hs-CRP and fasting insulin were higher in the ASF group. Serum fatty acid subtype arachidonic acid (AA) was higher in the ASF group. However, alpha-linolenic acid (ALA), linoleic acid (LA), eicosapentaenoic acid (EPA), and docosahexaenoic acid (DHA) were lower than the PBF group.

A total of 87.3% of the PBF group had an unhealthy lifestyle compared to 91.9% in the ASF group. Sedentary time was higher in the ASF group. However, BMI, BMI change, and body fat percentage were similar between groups. Also, 42.8% of those in the ASF group had a history of smoking compared to 39.4% in the PBF group. The proportion of respondents taking prescription pills for T2D was 31.6% and 40.2% in the ASF and PBF groups, respectively.

Table 2 shows the descriptive estimates stratified by T2D history. There were 286 cases of T2D, and of those, 52.1% were male. At the median, the T2D group was 17 years older, consumed less overall energy, and had a poorer body composition, higher serum triglycerides, lower HDL-C, lower fiber intake, higher intake of plant protein, lower intake of plant fats, higher intake of ASF fats, lower intake of ASF protein, higher glycohemoglobin, higher hS-CRP, higher insulin levels, higher fasting glucose, lower intake of cholesterol, lower intake of PUFAs and MUFAs, and higher sedentary time. However, those with T2D had a 14% lower intake of carbohydrates, a 12% lower saturated fat intake, lower LDL-C, lower EPA concentration, a lower dietary ω6FAs: ω3FAs ratio, and higher levels of AA, ALA, and DHA than those without T2D. The T2D group experienced a reduction in BMI in the past year, while non-diabetics saw a slight increase. Approximately 41.3% of those with T2D reported a smoking history, compared to 41.0% in non-diabetics. Of diabetic smokers, 15.6% were current smokers and 25.7% were former smokers.

**Figure 2:**
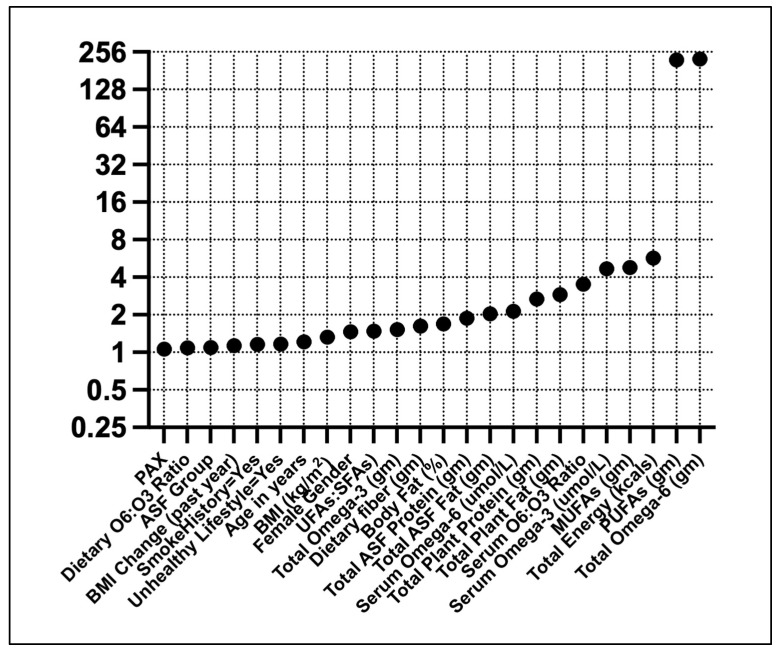
Variance inflation factors for T2D predictors. Predictors were standardized (Z) before the analysis.

Linear regression showed high VIFs for ω6FAs and PUFAs (Figure 2). Moderate VIFs were found for total energy, MUFAs, and serum ω3FAs.

The PCA resulted in 10 components extracted, explaining over 75% of the variance in T2D history (Table 3). Component 1 explained nearly 20% of the variance and was influenced primarily by high unsaturated fatty acids and high total energy intake. Component 2 explained nearly 9% of the variance, with a high factor loading from the female gender. A strong factor loading on Component 3 from the PBF dietary pattern explained 8.4%. Component 4 explained 7.1% of the variance with a strong negative loading from serum ω6FAs: ω3FAs ratio and a strong positive loading from serum ω3 FAs. Component 5 was influenced by higher levels of plant-based fats and proteins and lower ratios of dietary ω6: ω3 FAs, accounting for 6.3% of the variance. Component 6 had strong loadings of age, smoking history, an unhealthy lifestyle, and a negative loading of BMI change in the past year. Component 7 had high loadings of animal-sourced fat and protein. Component 8 was influenced by poor body composition, a recent BMI increase, and an unhealthy lifestyle. The ratio of UFs: SFAs loaded strongly on Component 9. Lastly, Component 10 was primarily influenced by lower ratios of physical activity to sedentary time.

**Figure 3:**
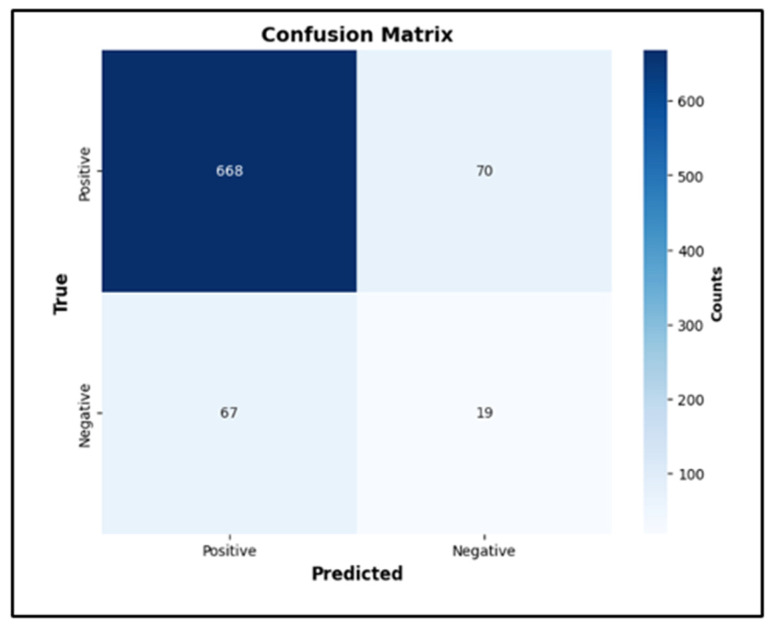

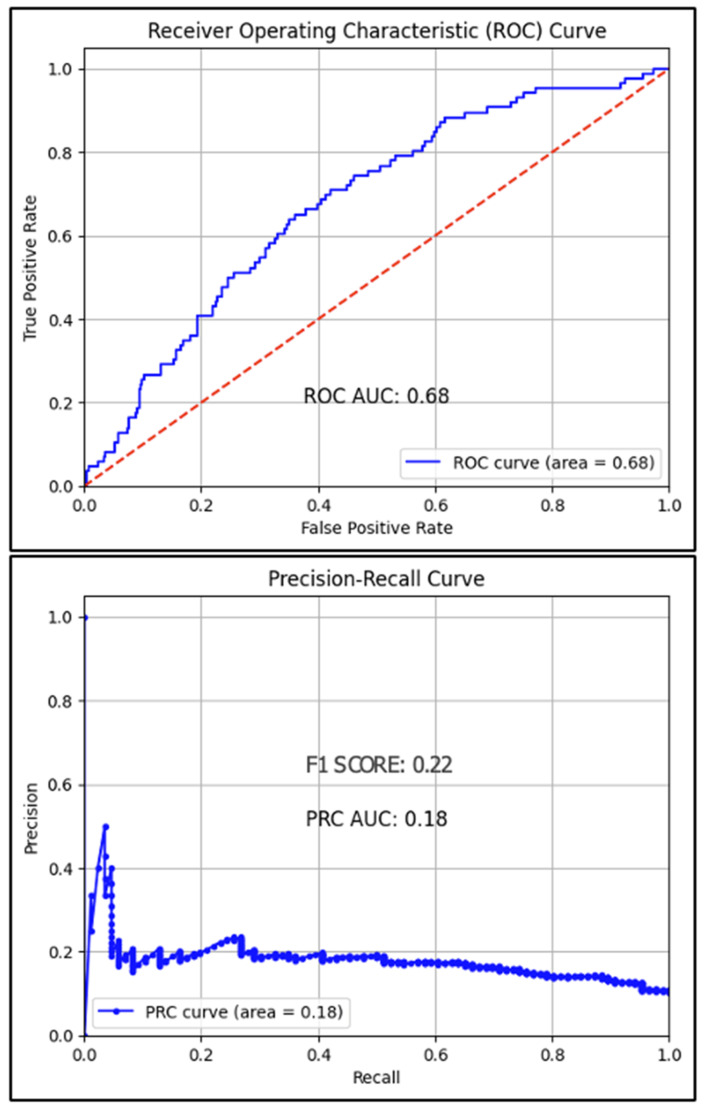
Confusion matrix (**top**), ROC curve (**middle**), and PRC (**bottom**) curve for the XGBoost classifier, red line: Reference Line.

The best hyperparameters for the XGBoost classifier included a gamma value of 0.05, a learning rate of 0.2, a max depth of 7, n-estimators of 150, and a subsample of 0.5. The mean accuracy across the 10-fold cross-validation was 94.3%. The AUROC was 68%, with an overall accuracy of 83.4% and an F1 score of 22% (Figure 3). The AUPRC for the T2D-positive class was 18%.

**Figure 4:**
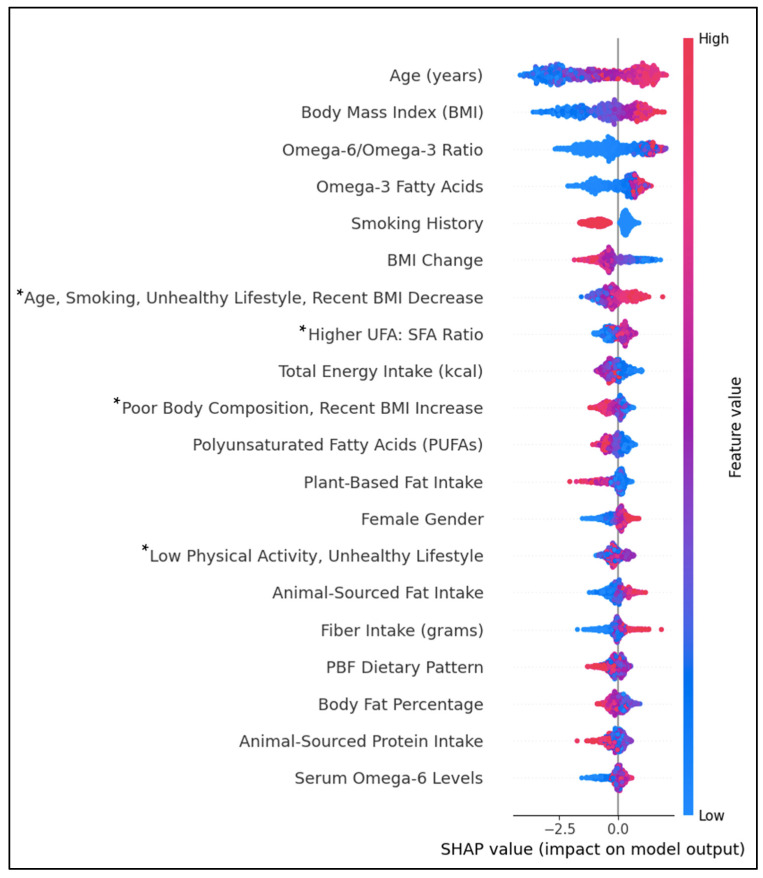
SHAP beeswarm plot ranking the top 20 features by their importance in predicting T2D history. Features on the *y*-axis increase in magnitude from left to right. Pink data points have a positive impact on prediction; blue data points have a negative impact on prediction. * PCA-engineered feature; data points represent regression factor scores. Higher scores indicate a stronger association with the feature.

Figure 4 shows the top individual and lifestyle predictors by feature importance. Positively associated predictors of T2D were age, BMI, the dietary ω6FAs: ω3FAs ratio, ω3FAs, female gender, ASF fat intake, fiber intake, and serum ω6FAs. Individual predictors inversely associated with T2D were smoking history, BMI change, PUFAs, PBF fat intake, and ASF protein intake. Diet–lifestyle features positively associated with T2D prediction were ‘Age, Smoking, Unhealthy Lifestyle, and Recent BMI Decrease’ and ‘Higher UFA: SFA Ratio’. In contrast, lifestyle features ‘Poor Body Composition, Recent BMI Increase’ and the PBF dietary pattern were negatively associated. The associations between body fat percentage and ‘Low Physical Activity, Unhealthy Lifestyle’ features were inconclusive. Predictive values for some features, including the PBF dietary pattern and body fat percentage, had substantial overlap between positive and negative impacts on prediction.

## 4. Discussion

This cross-sectional investigation observed complex dietary, lifestyle, and body composition patterns associated with T2D. High multicollinearity measures for UFAs confounded the relationship between dietary patterns and T2D history. Supporting established links, age, BMI, and an unhealthy lifestyle were among the top predictors of T2D history, emphasizing the role of age and lifestyle in disease progression [27,28]. However, the effect of smoking on T2D was unclear. Although an unhealthy lifestyle, defined by obesity, a marked recent increase in BMI, low physical activity, or smoking history was associated with T2D, smoking history as an individual predictor was inversely associated. Evidence suggests a complex relationship between smoking and T2D risk, with smoking contributing to a relatively higher risk of prediabetes and an increased risk of T2D following smoking cessation that gradually declines [29]. The increased risk after smoking cessation may be due to cumulative exposure to smoking or the result of weight gain following cessation. In the current study, smoking history included current and former smokers, so it is likely that the increased risk in those with and without a smoking history was exacerbated by unhealthy levels of body fat. Moreover, the prevalence of higher BMI and body fat percentage, despite recent BMI decreases, and the higher proportion of former smokers in the T2D group support the theory of weight gain following smoking cessation.

There was no clear link between either the ASF or PBF dietary pattern and T2D history. Despite the higher prevalence of T2D in the ASF group, higher levels of animal protein were negatively associated with T2D. This link is likely due to the strong link between ASF patterns and unhealthy lifestyle factors rather than the inclusion of ASFs. Conversely, diets higher in plant fats were inversely associated with T2D, like the studies mentioned previously. High amounts of circulating amino acids, specifically branched-chain amino acids (BCAAs), found in animal protein sources have been linked to hyperinsulinemia and impaired glucose uptake via the activation of the mammalian target of rapamycin (mTOR) pathway, which promotes protein synthesis and lipogenesis [30]. However, the effects of amino acid types on insulin action and glucose tolerance remain unclear. Recent clinical evidence suggests that the mTOR pathway is not activated with isocaloric, non-energy-restrictive substitutions of fat for animal or plant protein [31]. This may explain the attenuated associations between total and animal-sourced protein and increased risk of T2D or insulin resistance with an adjustment for body composition or total energy in several studies [32,33,34]. In contrast, one study found an association between animal-sourced and total protein intakes in obese women compared to men [35]. However, adjustment for previous chronic disease conditions weakened this relationship.

Evidence suggests that age, muscle mass, and adiposity have independent effects on insulin resistance [36,37,38]. In men, age appears to be an independent predictor of insulin resistance, while in females, increases in age-related adiposity drive insulin resistance [37]. In one study of over 100 post-menopausal women, lean body mass and visceral fat independently predicted insulin levels and hs-CRP, an inflammatory marker associated with increased CVD risk [36]. We found a similar result in the PCA, with female gender and body fat percentage loading strongly onto Component 2, accounting for 8.9% of the variance in T2D outcomes.

The difference in BCAA content between animal-sourced and plant-based protein sources may explain the association between protein type and insulin resistance, especially in older, overweight individuals. Animal protein consumption has been linked to higher muscle mass index (muscle mass (kg)/height (m)^2^) through higher muscle protein synthesis compared to plant protein [39]. However, animal protein intake and higher muscle mass may promote insulin resistance as age- and adiposity-related inflammation increase. In a cross-sectional study of older adults, insulin resistance measured by HOMA-IR was associated with animal protein intake, but only in those with a higher muscle mass index and higher body fat. In contrast, plant protein was inversely associated with muscle mass index and insulin resistance [38]. Importantly, however, a higher muscle mass index was inversely associated with body fat and chronic disease. The loss of type I muscle fibers, which are dense in mitochondria and have a high oxidative capacity, may also play a role in the development of T2D via increased fat deposition [40]. Our results support these effects. The ASF group had higher insulin levels and BMI but slightly lower body fat, which indicates relatively higher muscle mass. These findings suggest a complex relationship between age, diet, body composition, and insulin dysregulation.

A higher ratio of dietary ω6FAs: ω3FAs and higher serum ω6FAs predicted T2D. Evidence on the effects of UFAs on T2D or metabolic markers is unclear [4,41,42,43]. A meta-analysis of 10 cohort studies showed that ω3FA consumption was positively associated with T2D, exhibiting an inverted U-shape relationship [37]. In another meta-analysis of 83 randomized controlled trials, increasing ω3FAs, ω6FAs, or total PUFA had little or no effect on preventing or treating newly diagnosed T2D [44]. In the Prospective Metabolism and Islet Cell Evaluation (PROMISE) longitudinal study that included 477 participants with 6 years of follow-up, total non-esterified fatty acids (NEFAs) independently predicted decreased beta cell function [45]. No individual NEFAs had a positive influence on insulin sensitivity except for EPA. Similarly, the ASF group and T2D group had a higher overall intake of fats, including plant sources. This may be evidence of an adverse effect of overconsumption of UFAs despite a similar ratio of UFAs: SFAs to that of the PBF group.

Other research suggests that the relative dose of ω3FAs and long-term consumption may mediate this relationship [37,41]. Using NHANES data from 2005 to 2020, Jiang et al. observed that higher amounts of specific subtypes of MUFAs (16:1, 18:1, and 20:1) and PUFAs (18:2 and18:3) were related to reduced T2D risk [46]. Furthermore, only the highest intakes of PUFA subtypes 20:5 (EPA) and 22:5 (DPA) were associated with lower risk. In the current study, like Jiang et al., the T2D group had lower intakes of EPA, and the ASF group had lower intakes of EPA and DHA [46]. Conversely, the SHAP plot revealed an association between very high levels of ω3FAs and T2D, which may be due to secondary prevention efforts by diabetics to increase ω3FA levels.

In the current study, the ratio of serum ω6FAs: ω3FAs was over 13 for both groups. However, the ratios associated with improved metabolic health are 4–5:1 [24,25,47]. Although there is no consensus on the optimal ratios, some evidence suggests that the serum and dietary ω6FAs: ω3FAs ratio may predict T2D [24,25]. Modern diets have increased the availability of ω6FAs compared to ω3FAs, which has implications for metabolic health. ω6FAs are converted to AA, which may have inflammatory and thrombotic effects, whereas ω3FAs are converted to EPA and DHA, which exert anti-inflammatory effects [48]. ω6FAs may also contribute to obesity by increasing triglyceride concentration via increased cell membrane permeability, while ω3FAs have the opposite effect [49]. This is supported by several investigations showing a positive association between relatively higher amounts of ω3FAs and improved insulin sensitivity, fasting glucose, insulin, hs-CRP levels, and fitness measures [24,25,26].

The use of the ω6FAs:ω3FAs ratio has been questioned recently, however, due to the need for more reporting of specific subtypes used in the ratio [50]. Another criticism is that using a ratio does not consider the absolute amounts of each UFA despite evidence of a dose–response effect of specific UFAs in reducing T2D risk [26,46,50]. Furthermore, some evidence suggests that the lack of ω3FAs, not the relative increases in ω6FAs, is proinflammatory [50].

In another study in children and older adults that combined multiple cross-sectional and longitudinal studies, a lower ω6FAs:ω3FAs ratio was associated with higher HEI scores, indicating higher diet quality [47]. Still, participants across age groups did not meet the recommended amounts of EPA and DHA. Our study supports this as a higher ratio of ω6FAs:ω3FAs was predictive of T2D. Interestingly, higher ratios of UFAs:SFAs were predictive of T2D, despite the HEI standard of ≥2.5 for optimal FA consumption. Again, this association may be due to attempts by diabetics to improve the quality of their FA intake. Further investigation is needed to clarify the causal relationships between fatty acid subtypes and T2D.

Pancreatic beta cell function and their survival are related to the carbon chain length and degree of FA saturation, with long-chain SFAs (>12 carbons) inducing cytotoxicity [51]. However, in vitro studies have shown improvements in beta cell function after exposure to UFAs [52]. Some epidemiological evidence suggests that food preparation practices confound the relationship between UFAs and T2D. For example, Qian observed a 50% increase in T2D risk with higher consumption of fried plant-based MUFAs than animal-sourced MUFAs [53]. Deep frying typically involves using long-chain (18 carbons) plant oils. However, deep frying with low smoke point oils, especially with repeated use, increases oxidation and the production of SFAs and trans fats and impairs beta cell function [50,51].

Noteworthy observations from this study highlight the importance of lifestyle modification in preventing and treating T2D. First is the impact of BMI and unhealthy lifestyles on T2D outcomes. A recent investigation showed that just 14 days of reduced physical activity led to increases in insulin resistance and total body fat and decreases in limb lean mass [54]. Countless clinical trials representing the standard care model, which includes increasing physical activity and caloric restriction, have demonstrated effectiveness in reducing T2D risk and improving T2D and cardiometabolic markers [55]. Next is the consumption of nearly 135 more calories in the ASF group at the median despite being matched on total energy, BMI, and physical activity index. The link between T2D and energy surplus is inexorable, and recent attempts have been made to define T2D as an energy surplus disease [56]. Lack of dietary planning could lead to reduced dietary quality and over-consumption of calorically dense foods, including animal-sourced foods or processed foods from any source, increasing the risk of T2D. Caloric restriction, however, is a powerful tool in managing T2D. In a network meta-analysis of 18 studies on caloric restriction methods, intermittent fasting and continuous energy restriction methods improved HbA1c, body weight, and BMI compared to traditional diets [57].

Although dietary interventions for T2D management have been studied extensively, the best dietary pattern has yet to be identified. A recent meta-analysis of 56 trials comparing nine different dietary patterns showed that all dietary patterns were effective in reducing HbA1c and fasting glucose compared to the control, with a low-carbohydrate diet, Mediterranean diet, and Paleolithic diet among the most effective [58]. These dietary patterns share common characteristics favorable to metabolic profiles such as energy balance, higher UFA content, higher protein, higher intake of fruits and vegetables, minimally processed foods, and higher intakes of fiber.

### 4.1. Strengths

This is the first study incorporating XGBoost analysis to address the relationship between specific dietary patterns and T2D risk. We found that lifestyle variables confounded the relationship between nutrients from animal and plant sources and T2D history. By matching dietary patterns on common confounders, our study provides insights into how lifestyle and nutrient intake contexts influence T2D risk, offering a novel perspective on the interplay between diet and metabolic health. Although counter-matching has traditionally been employed to improve case–control efficiency, our study differs in that it uses propensity score matching to isolate dietary exposure groups for analysis of T2D predictors. The results of this study align with other studies employing ML methods for predicting T2D that found a high impact of age, body composition, and lifestyle variables on T2D [18,19,20,21]. However, the sets of predictors used across studies differed, which is likely to change feature importance. It is important to emphasize that researchers should consider the aims, sample characteristics, and sets of predictors used when comparing models. To our knowledge, this is the first study to employ XGBoost on an NHANES sample with PCA-engineered variables depicting diet–lifestyle patterns as additional features. Including PCA-engineered features allowed for the differentiation of importance for individual and interrelated predictors.

### 4.2. Limitations

The use of 24 h dietary recall and food-frequency questionnaires as a proxy for habitual intake is a weakness in this and many other dietary studies. We did not include sources of nutrients from beverages other than milk-based drinks and fruit juices, which does not account for all energy sources and prevents an in-depth examination of diet quality. However, the differences between the ASF and PBF groups were similar to those in other studies. Moreover, we did not account for other factors such as comorbidities, alcohol consumption, family history, or SES. However, due to their interrelatedness with other lifestyle factors, we did not deem it necessary to include all known/available background factors to illustrate issues with confounding. Prescription drug use could have biased serum marker estimates or skewed the associations between serum markers and T2D. Overall, prescription drug use was higher in the PBF group, suggesting increased rates of medical treatment and supporting the link between dietary patterns, healthy lifestyle bias, and disease outcomes. The cross-sectional design opens the possibility of reverse causality attribution, although many of our results are supported by prospective studies. Although we excluded individuals who reported being on a diet at the time of the survey, this questionnaire item may not account for all individuals who recently changed their lifestyle or dietary habits.

Furthermore, the results of this study may differ from future cross-sectional studies that include different age ranges. The XGBoost model achieved relatively high accuracy but low sensitivity, which may be due to the inclusion of interrelated predictors or the exclusion of other relevant predictors. However, our goal was not necessarily to find the best T2D predictors but to explore the importance of lifestyle and dietary factors in T2D history. Future studies should prospectively analyze the interactions of pre-existing metabolic conditions, nutrient intake thresholds, and T2D incidences to clarify the relationships further and propose new guidelines for management and prevention purposes.

### 4.3. Practical Implications

Although randomized controlled interventions significantly reduce bias and serve to isolate causal predictors, they are limited by funding, the extent to which nutrients and other physiological-influencing behaviors can be controlled, lack of long-term follow-up, and diet adherence issues, to name a few. While often having high statistical power, cross-sectional studies are often limited by retrospective design, lack of control variables and groups, lack of causal inference, and confounding due to unknown or uncollected variables. Despite these shortcomings, the wide availability (often publicly available) and high sample size of cohort-based datasets make these data sources readily accessible and have utility in providing insights on otherwise complex subject matter. In conjunction with incorporating control variables, robust ML techniques may improve the signal-to-noise ratio compared to traditional predictive methods.

## 5. Conclusions

This study highlights the complex interplay between dietary and lifestyle patterns and their association with T2D history, emphasizing the significant impact of age, body composition, and dietary fatty acid composition. Although the animal-sourced food pattern had a higher prevalence of T2D, this was primarily explainable by unhealthy lifestyles, yet animal-sourced protein intake was inversely associated. The XGBoost algorithm clarified the importance of interrelated multifactorial predictors, underscoring the limitations of traditional approaches in addressing confounding. Future research in this area should establish robust ML techniques and include longitudinal designs that would help isolate the effects of dietary and lifestyle factors on chronic disease outcomes. This approach can refine dietary guidelines and preventive strategies for T2D.

## Figures and Tables

**Table 1:** Descriptive estimates by dietary pattern.

	PBF Pattern (n = 1373) T2D (9.5%)		ASF Pattern (n = 1373) T2D (11.3%)		Median Diff. (%) [ASF/PBF]
	Median	IQR	Median	IQR	
Age (years)	48.00	31.00	47.00	30.00	0.98
Alpha-Linolenic acid (18:3n–3) (μmol/L)	73.30	54.50	69.80	46.60	0.95
Arachidonic acid (20:4n–6) (μmol/L)	784.00	352.00	829.00	345.00	1.06
ASF MUFAs (gm)			5.22	7.43	
ASF PUFAs (gm)			2.60	4.12	
Total ASF protein (gm)			23.88	26.19	
Total ASF fat (gm)			14.64	19.62	
BMI Change (past year)	0.19	2.23	0.23	2.60	1.25
Body Mass Index (kg/m^2^)	27.10	8.70	27.80	8.30	1.03
Carbohydrate (gm)	243.59	132.84	238.31	151.00	0.98
Cholesterol (mg)	220.00	256.00	300.00	301.00	1.36
Total dietary omega–3 (gm)	0.09	0.24	0.24	0.45	2.74
Total dietary omega–6 (gm)	15.08	12.77	16.74	11.95	1.11
Dietary O6:O3 ratio	131.24	431.06	61.46	145.56	0.47
Dietary fiber (gm)	16.90	12.70	15.20	12.60	0.90
Direct HDL-Cholesterol (mg/dL)	53.00	22.00	52.00	22.00	0.98
Docosahexaenoic acid (22:6n–3) (μmol/L)	152.00	88.00	140.00	81.00	0.92
Eicosapentaenoic acid (20:5n–3) (μmol/L)	53.40	42.70	51.80	51.40	0.97
Energy (kcal)	2028.00	1046.00	2161.00	1191.00	1.07
Fasting glucose (mg/dL)	100.00	14.00	100.00	15.00	1.00
Glycohemoglobin (%)	5.40	0.50	5.40	0.60	1.00
hs-CRP (mg/L)	1.50	3.50	1.80	3.4	1.20
Insulin (μU/mL)	8.65	8.67	9.03	9.23	1.04
LDL-cholesterol (mg/dL)	108.00	46.00	110.00	50.00	1.02
Linoleic acid (18:2n–6) (μmol/L)	3410.00	1220.00	3220.00	1060.00	0.94
Minutes sedentary activity	420.00	300.00	360.00	300.00	0.86
PAX	0.00	0.00	0.00	0.00	
PBF MUFAs (gm)	1.68	4.39	3.12	6.62	1.86
PBF PUFAs (gm)	1.23	3.08	1.91	5.37	1.55
Total plant protein (gm)	4.19	7.37	6.25	12.24	1.49
Total plant fat (gm)	5.22	12.08	9.54	18.82	1.83
Total kcals from PBFs (%)	9.0	13.0	0.00	10.0	0.00
Total kcals from ASFs (%)			14.71	17.4	
Protein (gm)	73.68	46.13	86.44	54.70	1.17
Serum omega–6 (μmol/L)	4453.10	1409.80	4416.90	1449.30	0.99
Serum omega–3 (μmol/L)	341.65	205.00	344.17	180.42	1.01
Serum omega–6: omega–3 ratio	13.21	5.32	13.57	5.34	1.03
Total cholesterol (mg/dL)	186.00	54.00	186.00	57.00	1.00
Total fat (gm)	75.79	50.07	82.83	55.85	1.09
Total monounsaturated fatty acids (gm)	26.61	18.89	28.64	20.21	1.08
Total body fat (%) [DEXA]	32.00	12.20	31.70	13.40	0.99
Total polyunsaturated fatty acids (gm)	16.91	14.07	18.78	13.59	1.11
Total saturated fatty acids (gm)	24.43	18.54	26.78	21.31	1.10
Total daily recreational physical activity (min)	0.00	0.00	0.00	0.00	
Triglyceride (mg/dL)	94.00	78.00	90.00	72.00	0.96
UFAs:SFAs ratio	1.83	0.99	1.83	0.93	1.00

**Table 2:** Descriptive estimates by T2D status.

	Non-Diabetics (n = 2460)		Diabetics (n = 286)		Median Diff. (%) [T2D/Non-T2D]
	Median	IQR	Median	IQR	
Age (years)	46.00	30.00	63.00	16.00	1.37
Alpha-Linolenic acid (18:3n–3) (μmol/L)	72.00	49.30	75.50	59.00	1.05
Arachidonic acid (20:4n–6) (μmol/L)	797.00	352.00	886.00	307.00	1.11
ASF MUFAs (gm)	5.20	7.42	5.69	8.26	1.09
ASF PUFAs (gm)	2.57	4.11	3.34	4.30	1.30
Total ASF protein (gm)	24.04	26.50	22.51	21.72	0.94
Total ASF fat (gm)	14.63	19.62	15.56	20.13	1.06
Total kcals from ASFs (%)	15.00	17.00	16.00	19.00	1.07
BMI Change (past year)	0.26	2.34	−0.60	2.98	−2.33
Body Mass Index (kg/m^2^)	27.20	8.40	31.10	8.00	1.14
Carbohydrate (gm)	244.18	143.49	209.18	119.75	0.86
Cholesterol (mg)	249.00	284.00	232.00	289.00	0.93
Total dietary omega–3 (gm)	0.16	0.34	0.17	0.38	1.02
Total dietary omega–6 (gm)	16.15	12.40	13.55	11.34	0.84
Dietary O6:O3 ratio	90.32	276.07	68.67	261.12	0.76
Dietary fiber (gm)	16.20	12.70	14.90	10.60	0.92
Direct HDL-Cholesterol (mg/dL)	52.00	22.00	47.00	19.00	0.90
Docosahexaenoic acid (22:6n–3) (μmol/L)	143.00	79.00	152.00	78.00	1.06
Eicosapentaenoic acid (20:5n–3) (μmol/L)	53.40	47.40	51.90	53.30	0.97
Energy (kcal)	2101.00	1141.00	1781.00	1036.00	0.85
Fasting Glucose (mg/dL)	99.00	13.00	134.00	62.00	1.35
Glycohemoglobin (%)	5.40	0.50	6.80	1.80	1.26
hs-CRP (mg/L)	1.60	3.40	2.40	4.90	1.5
Insulin (μU/mL)	8.65	8.76	11.47	11.19	1.33
LDL-cholesterol (mg/dL)	110.00	48.00	100.00	54.00	0.91
Linoleic acid (18:2n–6) (μmol/L)	3320.00	1100.00	3410.00	1390.00	1.03
Minutes sedentary activity	360.00	300.00	420.00	240.00	1.17
PAX	0.00	0.00	0.00	0.00	
PBF MUFAs (gm)	1.94	5.22	1.36	4.16	0.70
PBF PUFAs (gm)	1.38	3.50	1.24	3.10	0.90
Total plant protein (gm)	4.62	8.69	4.83	7.50	1.05
Total plant fat (gm)	6.32	13.97	4.00	12.19	0.63
Total kcals from PBFs (%)	0.05	0.14	0.05	0.14	1.02
Protein (gm)	81.27	51.96	68.14	40.53	0.84
Serum omega–6 (μmol/L)	4424.00	1426.30	4545.20	1706.10	1.02
Serum omega–3 (μmol/L)	344.17	185.80	336.54	175.19	0.98
Serum omega–6:omega–3 ratio	13.36	5.24	13.29	6.52	0.99
Total Cholesterol (mg/dL)	186.00	55.00	171.00	63.00	0.92
Total fat (gm)	79.70	53.87	69.41	45.09	0.87
Total monounsaturated fatty acids (gm)	27.64	19.84	23.94	19.01	0.87
Total body fat (%) [DEXA]	31.50	12.70	39.10	9.50	1.24
Total polyunsaturated fatty acids (gm)	18.21	13.64	15.52	12.43	0.85
Total saturated fatty acids (gm)	25.51	20.17	22.51	17.03	0.88
Total daily recreational physical activity (min)	0.00	0.00	0.00	0.00	
Triglyceride (mg/dL)	91.00	68.00	114.00	102.00	1.25
UFAs: SFAs ratio	1.82	0.98	1.89	0.86	1.03

**Table 3:** PCA pattern matrix for diet and lifestyle features.

Component	1	2	3	4	5	6	7	8	9	10
Variance Explained (%)	19.8	8.9	8.4	7.1	6.3	6.0	5.6	4.7	4.2	4.0
	Higher Total Energy and UFAs	Female Gender	PBF Dietary Pattern	Lower SerumO6:O3 Ratio, Higher sO3	Higher PBF Fat and Protein, Lower Dietary O6:O3	Age, Smoking, Unhealthy Lifestyle, Recent BMI Decrease	ASF Fat and Protein	Poor Body Composition, Recent BMI Increase	Higher UFA:SFA Ratio	Low Physical Activity, Unhealthy Lifestyle
Total Omega-6 (gm)	0.89	0.03	−0.02	−0.05	0.05	0.01	0.03	0.02	0.18	0.01
PUFAs (gm)	0.89	0.03	−0.02	−0.05	0.05	0.01	0.04	0.01	0.18	0.01
MUFAs (gm)	0.89	−0.04	−0.03	−0.01	0.02	0.00	0.05	0.00	−0.10	−0.02
Total Energy (kcals)	0.85	−0.11	−0.01	−0.01	0.07	−0.03	0.07	−0.01	−0.23	−0.03
Total Fiber (gm)	0.63	−0.07	0.14	0.11	0.10	−0.03	−0.12	−0.03	0.04	−0.16
Male Gender	0.01	−0.98	0.00	0.00	0.00	0.01	0.00	0.02	0.02	−0.01
Female Gender	−0.01	0.98	0.00	0.00	0.00	−0.01	0.00	−0.02	−0.02	0.01
PBF Dietary Pattern	−0.01	0.00	1.00	0.00	0.01	0.00	0.01	0.00	0.00	0.01
ASF Dietary Pattern	0.01	0.00	−1.00	0.00	−0.01	0.00	−0.01	0.00	0.00	−0.01
Total Serum Omega-3 (μmol/L)	−0.01	0.00	0.00	0.96	−0.01	0.00	0.00	−0.01	0.00	0.01
Total Serum Omega-6 (μmol/L)	0.29	0.10	−0.05	0.64	−0.08	−0.02	−0.06	−0.05	−0.10	0.24
Serum O6:O3 Ratio	0.22	0.06	−0.04	−0.75	−0.04	−0.03	−0.08	−0.03	−0.07	0.17
Total Plant Protein (gm)	0.04	−0.01	0.07	0.02	0.88	−0.02	−0.08	−0.02	−0.02	−0.01
Total Plant Fat (gm)	0.12	0.03	0.01	−0.03	0.86	0.02	−0.03	0.00	−0.03	0.02
Total Omega-3 (gm)	−0.05	0.01	−0.16	−0.03	0.62	−0.01	0.27	0.02	0.20	0.01
Age (years)	−0.06	−0.02	−0.03	0.13	−0.04	0.67	−0.11	0.12	0.18	0.00
Smoker	0.06	−0.21	0.01	−0.04	0.00	0.55	0.06	0.09	−0.17	0.22
Unhealthy Lifestyle	−0.01	0.01	0.00	−0.03	0.03	0.45	0.02	0.30	−0.14	0.45
BMI Change (past year)	0.01	−0.05	0.00	0.06	0.00	−0.63	−0.04	0.42	0.01	0.31
Total ASF Protein (gm)	−0.02	−0.01	0.01	0.04	−0.01	−0.01	0.90	0.00	0.01	−0.01
Total ASF Fat (gm)	0.08	0.02	0.02	−0.01	−0.03	0.00	0.89	0.01	−0.02	0.02
BMI (kg/m^2^)	0.04	−0.06	−0.02	−0.03	−0.02	−0.01	0.03	0.89	0.01	−0.08
Body Fat (%)	−0.09	0.48	0.03	0.04	0.03	0.09	−0.03	0.58	0.00	−0.06
UFAs:SFAs	0.07	−0.04	0.00	0.00	0.01	0.01	−0.01	0.01	0.92	0.05
PAX	0.09	−0.01	−0.03	0.01	−0.02	0.04	−0.01	0.13	−0.09	−0.84
Dietary O6:O3 Ratio	0.38	0.09	0.16	−0.03	−0.41	0.04	−0.03	−0.02	0.18	−0.02

Pattern matrix for ten principal components, explaining over 75% of the variance in T2D history. The table values are regression coefficients that reflect the unique contribution of each variable to the component. Components were rotated via direct oblimin (Δ = 0). Darker colors indicate a stronger coefficient between the predictor and the component. Blue = positive association with the component; red = negative association with the component.

## Data Availability

The data used throughout this study were derived from the following resources available in the public domain: https://www.cdc.gov/nchs/nhanes/?CDC_AAref_Val=https://www.cdc.gov/nchs/nhanes/index.htm accessed on 18 December 2024.

## Supplementary Materials

The following supporting information can be downloaded at: https://www.mdpi.com/article/10.3390/jcm14020458/s1, Table S1. Aggregated and Derived Variables.

## Abbreviations

ASFs	animal-sourced foods
AUPRC	area under the precision-recall curve
AUROC	area under the receiver operating characteristic curve
BMI	body mass index
FAs	fatty acids
HEI	healthy eating index
HS-CRP	high-sensitivity c-reactive protein
ML	machine learning
MUFAs	monounsaturated fatty acids
PA	physical activity
PAX	ratio of total physical activity to sedentary time
PBFs	Plant-based foods
PUFAs	polyunsaturated fatty acids
RCT	randomized controlled trial
SFAs	saturated fatty acids
T2D	Type 2 diabetes
ω3FAs	dietary omega-3 fatty acids
ω6FAs	dietary omega-6 fatty acids
ω6FAs	ω3FAs ratio of omega-6 to omega-3 fatty acids
UFAs	unsaturated fatty acids
