# Correction: Eckart, A.C.; Sharma Ghimire, P. Exploring Predictors of Type 2 Diabetes Within Animal-Sourced and Plant-Based Dietary Patterns with the XGBoost Machine Learning Classifier: NHANES 2013–2016. *J. Clin. Med.* 2025, *14*, 458

**DOI:** 10.3390/jcm14217600

**Published:** 2025-10-27

**Authors:** Adam C. Eckart, Pragya Sharma Ghimire

**Affiliations:** Department of Health and Human Performance, Kean University, Union, NJ 07083, USA; pghimire@kean.edu

## Text Correction

In the original publication [1], there were mistakes in the Materials and Methods Section and paragraph 7 of the Results Section. In the Materials and Methods Section, the original wording may be interpreted as describing supervised PCA, in which Type 2 Diabetes (T2D) status served as the dependent variable. The authors clarify that, in Section 2.5 (Statistical Analyses), unsupervised principal component analysis (PCA) was conducted to derive new diet–lifestyle features. This clarification is essential to ensure an accurate understanding of the analytic approach, as the use of an unsupervised method indicates that component extraction was independent of T2D outcome status, thereby avoiding potential bias in feature generation.

In addition, in the first sentence of paragraph 7 in the Results Section, the reported explained variance from the PCA model may be interpreted as describing variance in the T2D outcome. As the PCA conducted was unsupervised, the explained variance in both locations refers to the proportion of total variance accounted for by the extracted principal components, not variance in T2D status. These corrections do not affect the study’s results, statistical outputs, or conclusions. All analyses and findings remain unchanged.

## Corrections Have Been Made to the Materials and Methods Section and Results Section:

### 2.5. Statistical Analyses

To examine multicollinearity, linear regression was used to derive each predictor’s variance inflation factor (VIF). We used unsupervised principal component analysis (PCA) to analyze diet–lifestyle patterns independently of disease status. Principal components were rotated via direct oblimin (∆ = 0) to maximize interpretability. Regression factor scores from each component were added as new features. The variable pattern loadings on each component were characterized according to the strength of partial correlations for each predictor in the pattern matrix.

### Results Section Paragraph 7

The PCA resulted in 10 components being extracted, explaining over 75% of the variance (Table 3). Component 1 explained nearly 20% of the variance and was influenced primarily by high unsaturated fatty acids and high total energy intake. Component 2 explained nearly 9% of the variance, with a high factor loading from the female gender.

## Error in Tables

There was an error in the column heading “Median Diff. (%)” in Tables 1 and 2, which implies that the values represent percentage differences between the nutrient intakes of the groups compared. The reported values are, in fact, proportions (decimal form). The column heading will be revised to indicate proportions rather than percentages to ensure accurate representation of the data. The corrected Table 1 and Table 2 appear below.

### Table Legend

There was an error in the footer for Table 3; the reported explained variance from the PCA model may be interpreted as describing variance in the T2D outcome. As the PCA conducted was unsupervised, the explained variance refers to the proportion of total variance accounted for by the extracted principal components, not variance in T2D status. The correct legend appears below.

Pattern matrix for ten principal components, explaining over 75% of the variance in T2D predictors. The table values are regression coefficients that reflect the unique contribution of each variable to the component. Components were rotated via direct oblimin (∆ = 0). Darker colors indicate a stronger coefficient between the predictor and the component. Blue = positive association with the component; red = negative association with the component.

## Error in Figure

There was an error in the Confusion Matrix as published in Figure 3. The labels for the “Positive” and “Negative” classes were inadvertently swapped in the previous version of the figure. The corrected figure now displays the proper class labels for both the True and Predicted axes, accurately representing the performance of the XGBoost classification model. The corrected Figure 3 appears below.

The authors state that the scientific conclusions are unaffected. These corrections were approved by the Academic Editor. The original publication has also been updated.

## Figures and Tables

**Figure 3:**
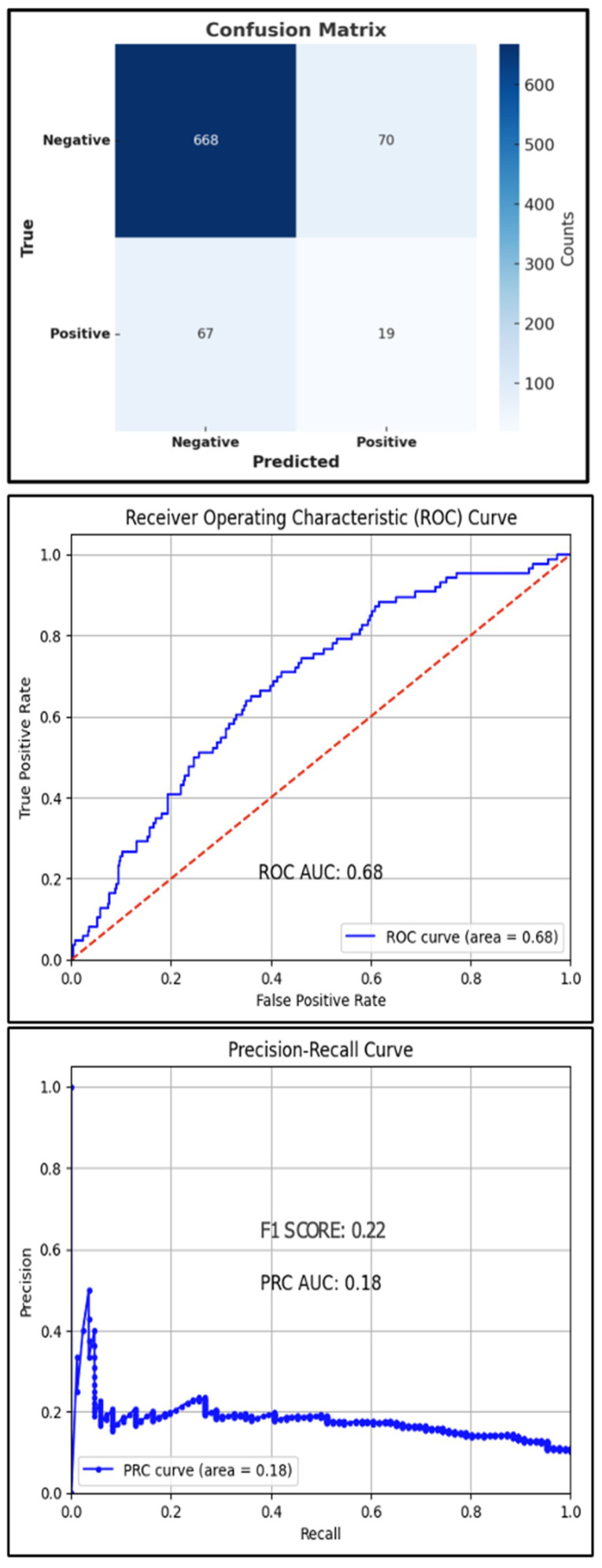
Test set confusion matrix (**top**), ROC curve (**middle**), and PRC (**bottom**) curve for the XGBoost classifier, red line: Reference Line.

**Table 1:** Descriptive estimates by dietary pattern.

	PBF Pattern (n = 1373) T2D (9.5%)		ASF Pattern (n = 1373) T2D (11.3%)		Median Diff. [ASF ÷ PBF]
	Median	IQR	Median	IQR	
Age (years)	48.00	31.00	47.00	30.00	0.98
Alpha-Linolenic acid (18:3n–3) (μmol/L)	73.30	54.50	69.80	46.60	0.95
Arachidonic acid (20:4n–6) (μmol/L)	784.00	352.00	829.00	345.00	1.06
ASF MUFAs (gm)			5.22	7.43	
ASF PUFAs (gm)			2.60	4.12	
Total ASF protein (gm)			23.88	26.19	
Total ASF fat (gm)			14.64	19.62	
BMI Change (past year)	0.19	2.23	0.23	2.60	1.25
Body Mass Index (kg/m^2^)	27.10	8.70	27.80	8.30	1.03
Carbohydrate (gm)	243.59	132.84	238.31	151.00	0.98
Cholesterol (mg)	220.00	256.00	300.00	301.00	1.36
Total dietary omega-3 (gm)	0.09	0.24	0.24	0.45	2.74
Total dietary omega-6 (gm)	15.08	12.77	16.74	11.95	1.11
Dietary O6:O3 ratio	131.24	431.06	61.46	145.56	0.47
Dietary fiber (gm)	16.90	12.70	15.20	12.60	0.90
Direct HDL-Cholesterol (mg/dL)	53.00	22.00	52.00	22.00	0.98
Docosahexaenoic acid (22:6n–3) (μmol/L)	152.00	88.00	140.00	81.00	0.92
Eicosapentaenoic acid (20:5n–3) (μmol/L)	53.40	42.70	51.80	51.40	0.97
Energy (kcal)	2028.00	1046.00	2161.00	1191.00	1.07
Fasting glucose (mg/dL)	100.00	14.00	100.00	15.00	1.00
Glycohemoglobin (%)	5.40	0.50	5.40	0.60	1.00
hs-CRP (mg/L)	1.50	3.50	1.80	3.4	1.20
Insulin (μU/mL)	8.65	8.67	9.03	9.23	1.04
LDL-cholesterol (mg/dL)	108.00	46.00	110.00	50.00	1.02
Linoleic acid (18:2n–6) (μmol/L)	3410.00	1220.00	3220.00	1060.00	0.94
Minutes sedentary activity	420.00	300.00	360.00	300.00	0.86
PAX	0.00	0.00	0.00	0.00	
PBF MUFAs (gm)	1.68	4.39	3.12	6.62	1.86
PBF PUFAs (gm)	1.23	3.08	1.91	5.37	1.55
Total plant protein (gm)	4.19	7.37	6.25	12.24	1.49
Total plant fat (gm)	5.22	12.08	9.54	18.82	1.83
Total kcals from PBFs (%)	9.0	13.0	0.00	10.0	0.00
Total kcals from ASFs (%)			14.71	17.4	
Protein (gm)	73.68	46.13	86.44	54.70	1.17
Serum omega-6 (μmol/L)	4453.10	1409.80	4416.90	1449.30	0.99
Serum omega-3 (μmol/L)	341.65	205.00	344.17	180.42	1.01
Serum omega-6:omega-3 ratio	13.21	5.32	13.57	5.34	1.03
Total cholesterol (mg/dL)	186.00	54.00	186.00	57.00	1.00
Total fat (gm)	75.79	50.07	82.83	55.85	1.09
Total monounsaturated fatty acids (gm)	26.61	18.89	28.64	20.21	1.08
Total body fat (%) [DEXA]	32.00	12.20	31.70	13.40	0.99
Total polyunsaturated fatty acids (gm)	16.91	14.07	18.78	13.59	1.11
Total saturated fatty acids (gm)	24.43	18.54	26.78	21.31	1.10
Total daily recreational physical activity (mins)	0.00	0.00	0.00	0.00	
Triglyceride (mg/dL)	94.00	78.00	90.00	72.00	0.96
UFAs:SFAs ratio	1.83	0.99	1.83	0.93	1.00

**Table 2:** Descriptive estimates by T2D status.

	Non-Diabetics (n = 2460)		Diabetics (n = 286)		Median Diff. [T2D ÷ Non-T2D]
	Median	IQR	Median	IQR	
Age (years)	46.00	30.00	63.00	16.00	1.37
Alpha-Linolenic acid (18:3n–3) (μmol/L)	72.00	49.30	75.50	59.00	1.05
Arachidonic acid (20:4n–6) (μmol/L)	797.00	352.00	886.00	307.00	1.11
ASF MUFAs (gm)	5.20	7.42	5.69	8.26	1.09
ASF PUFAs (gm)	2.57	4.11	3.34	4.30	1.30
Total ASF protein (gm)	24.04	26.50	22.51	21.72	0.94
Total ASF fat (gm)	14.63	19.62	15.56	20.13	1.06
Total kcals from ASFs (%)	15.00	17.00	16.00	19.00	1.07
BMI Change (past year)	0.26	2.34	−0.60	2.98	−2.33
Body Mass Index (kg/m^2^)	27.20	8.40	31.10	8.00	1.14
Carbohydrate (gm)	244.18	143.49	209.18	119.75	0.86
Cholesterol (mg)	249.00	284.00	232.00	289.00	0.93
Total dietary omega-3 (gm)	0.16	0.34	0.17	0.38	1.02
Total dietary omega-6 (gm)	16.15	12.40	13.55	11.34	0.84
Dietary O6:O3 ratio	90.32	276.07	68.67	261.12	0.76
Dietary fiber (gm)	16.20	12.70	14.90	10.60	0.92
Direct HDL-Cholesterol (mg/dL)	52.00	22.00	47.00	19.00	0.90
Docosahexaenoic acid (22:6n–3) (μmol/L)	143.00	79.00	152.00	78.00	1.06
Eicosapentaenoic acid (20:5n–3) (μmol/L)	53.40	47.40	51.90	53.30	0.97
Energy (kcal)	2101.00	1141.00	1781.00	1036.00	0.85
Fasting Glucose (mg/dL)	99.00	13.00	134.00	62.00	1.35
Glycohemoglobin (%)	5.40	0.50	6.80	1.80	1.26
hs-CRP (mg/L)	1.60	3.40	2.40	4.90	1.5
Insulin (μU/mL)	8.65	8.76	11.47	11.19	1.33
LDL-cholesterol (mg/dL)	110.00	48.00	100.00	54.00	0.91
Linoleic acid (18:2n–6) (μmol/L)	3320.00	1100.00	3410.00	1390.00	1.03
Minutes sedentary activity	360.00	300.00	420.00	240.00	1.17
PAX	0.00	0.00	0.00	0.00	
PBF MUFAs (gm)	1.94	5.22	1.36	4.16	0.70
PBF PUFAs (gm)	1.38	3.50	1.24	3.10	0.90
Total plant protein (gm)	4.62	8.69	4.83	7.50	1.05
Total plant fat (gm)	6.32	13.97	4.00	12.19	0.63
Total kcals from PBFs (%)	0.05	0.14	0.05	0.14	1.02
Protein (gm)	81.27	51.96	68.14	40.53	0.84
Serum omega-6 (μmol/L)	4424.00	1426.30	4545.20	1706.10	1.02
Serum omega-3 (μmol/L)	344.17	185.80	336.54	175.19	0.98
Serum omega-6:omega-3 ratio	13.36	5.24	13.29	6.52	0.99
Total Cholesterol (mg/dL)	186.00	55.00	171.00	63.00	0.92
Total fat (gm)	79.70	53.87	69.41	45.09	0.87
Total monounsaturated fatty acids (gm)	27.64	19.84	23.94	19.01	0.87
Total body fat (%) [DEXA]	31.50	12.70	39.10	9.50	1.24
Total polyunsaturated fatty acids (gm)	18.21	13.64	15.52	12.43	0.85
Total saturated fatty acids (gm)	25.51	20.17	22.51	17.03	0.88
Total daily recreational physical activity (mins)	0.00	0.00	0.00	0.00	
Triglyceride (mg/dL)	91.00	68.00	114.00	102.00	1.25
UFAs:SFAs ratio	1.82	0.98	1.89	0.86	1.03
