# Supplementary material for: Exploring Predictors of Type 2 Diabetes Within Animal-Sourced and Plant-Based Dietary Patterns with the XGBoost Machine Learning Classifier: NHANES 2013–2016
Source: J Clin Med. 2025 Jan 13;14(2):458. doi: 10.3390/jcm14020458 (PMC11766419; doi:10.3390/jcm14020458)
Supplement: Supplementary file 1 [file jcm-14-00458-s001.zip › jcm-3378306-supplementary.pdf]

Supplementary Table S1. Aggregated and Derived Variables

NHANES Variable Code	Variable	Doc File	Derived Variables
WTMEC2YR	Full sample 2-year MEC exam weight	Demographic Variables and Sample Weights (DEMO)	4-year MEC exam weight
SDMVPSU	Masked variance pseudo-PSU		
SDMVSTRA	Masked variance pseudo-stratum		
RIDAGEYR	Age in years at screening		
RIAGENDR	Gender		
WTDRD1	Dietary day one sample weight	Dietary Interview - Individual Foods, First Day	4-year dietary day one sample weight
DR1IFDCD	USDA food code		ASF and PBF dietary patterns
WWEIA	What We Eat in America food codes		
DR1CCMTX	Combination food type	DR1IFF	Combination and non-combination food categories
DR1IKCAL	Energy (kcal)	DR1IFF	ASF protein<br>ASF fats<br>PBF protein<br>PBF fat<br>PUFAs from ASFs<br>PUFAs from PBFs<br>MUFAs from ASFs<br>MUFAs from PBFs
DR1IPROT	Protein (gm)		
DR1ICARB	Carbohydrate (gm)		
DR1IFIBE	Dietary fiber (gm)		
DR1ITFAT	Total fat (gm)		
DR1IMFAT	Total monounsaturated fatty acids (gm)		
DR1IPFAT	Total polyunsaturated fatty acids (gm)		
DR1ISFAT	Total saturated fatty acids (gm)		

DR1IP182	PFA 18:2 (Octadecadienoic) (gm)		
DR1IP183	PFA 18:3 (Octadecatrienoic) (gm)		
DR1IP184	PFA 18:4 (Octadecatetraenoic) (gm)		
DR1IP204	PFA 20:4 (Eicosatetraenoic) (gm)		
DR1IP205	PFA 20:5 (Eicosapentaenoic) (gm)		
DR1IP225	PFA 22:5 (Docosapentaenoic) (gm)		
DR1IP226	PFA 22:6 (Docosahexaenoic) (gm)		
DR1TKCAL	Energy (kcal)	Dietary Interview - Total Nutrient Intakes, First Day (DR1TOT)	
DR1TPROT	Protein (gm)		
DR1TCARB	Carbohydrate (gm)		
DR1TFIBE	Dietary fiber (gm)		
DR1TTFAT	Total fat (gm)		
DR1TSFAT	Total saturated fatty acids (gm)		UFA: SFA ratio
DR1TMFAT	Total monounsaturated fatty acids (gm)		Dietary omega-6: omega-3 ratio
DR1TSFAT	Total saturated fatty acids (gm)		
DR1TCHOL	Cholesterol (mg)		
DR1TP205	PFA 20:5 (Eicosapentaenoic) (gm)		

DR1TP226	PFA 22:6 (Docosahexaenoic) (gm)		
DR1TP182	PFA 18:2 (Octadecadienoic) (gm)		
DR1TP183	PFA 18:3 (Octadecatrienoic) (gm)		
DR1TP184	PFA 18:4 (Octadecatetraenoic) (gm)		
DR1TP204	PFA 20:4 (Eicosatetraenoic) (gm)		
DR1TP225	PFA 22:5 (Docosapentaenoic) (gm)		
BMXBMI	Body Mass Index (kg/m**2)	Body Measures (BMX)	Unhealthy lifestyle: BMI (≥30 kg/m²)
DXDTOPF	Total Percent Fat - DEXA		
DIQ010	Doctor told you have diabetes	Diabetes (DIQ)	
DIQ050	Taking insulin now		
DIQ070	Take diabetic pills to lower blood sugar		
PAD660	Minutes vigorous recreational activities		Unhealthy lifestyle: less than 30 minutes of daily recreational physical activity at any intensity<br><br>Total physical activity to sedentary ratio (PAX)
PAD675	Minutes moderate recreational activities		
PAD680	Minutes sedentary activity		
WHD050	Self-reported weight - 1 yr ago (pounds)	Weight History (WHQ)	BMI change (past year)<br><br>Unhealthy lifestyle: 1-SD increase in BMI within the past year
SMQ040	Do you now smoke cigarettes	Smoking - Cigarette Use (SMQ)	Unhealthy lifestyle: smoking history
SMQ050Q	How long since quit smoking cigarettes		

WTSAF2YR	Fasting Subsample 2 Year MEC Weight		4-year fasting MEC weight
LBDHDD	Direct HDL-Cholesterol (mg/dL)	Cholesterol - HDL (HDL)	
LBXTR	Triglyceride (mg/dL)	Cholesterol - LDL & Triglycerides (TRIGLY)	
LBDLDL	LDL-cholesterol (mg/dL)		
WTFAS2YR	Fatty Acid Subsample 2 Year Weight	Fatty Acids - Serum (FAS)	4-years fatty acid weight
LBXLNA	Linoleic acid (18:2n-6) (umol/L)		Serum omega-6: omega-3 ratio
LBXALN	alpha-Linolenic acid (18:3n-3) (umol/L)		
LBXGLA	gamma-Linolenic acid (18:3n-6) (umol/L)		
LBXSD1	Stearidonic acid (C18:4n-3) (umol/L)		
LBXED1	Eicosadienoic acid (20:2n-6) (umol/L)		
LBXHGL	homo-gamma-Linolenic acid(20:3n-6)(uM/L)		
LBXARA	Arachidonic acid (20:4n-6) (umol/L)		
LBXEPA	Eicosapentaenoic acid (20:5n-3) (umol/L)		
LBXDTA	Docosatetraenoic acid (22:4n-6) (umol/L)		
LBXDP3	Docosapentaenoic acid (22:5n-3) (umol/L)		
LBXDP6	Docosapentaenoic acid (22:5n-6) (umol/L)		
LBXDHA	Docosahexaenoic acid (22:6n-3) (umol/L)		

LBXGH	Glycohemoglobin (%)	Glycohemoglobin (GHB)	
LBXIN	Insulin (uU/mL)	Insulin (INS)	
LBXHSCRP	HS C-Reactive Protein (mg/L)	High-Sensitivity C-Reactive Protein (hs-CRP) (HSCRP)	
LBXGLU	Fasting Glucose (mg/dL)	Plasma Fasting Glucose (GLU)	
